# Antibodies to myelin oligodendrocyte glycoprotein in HIV-1 associated neurocognitive disorder: a cross-sectional cohort study

**DOI:** 10.1186/1742-2094-7-79

**Published:** 2010-11-17

**Authors:** Peter Lackner, Bettina Kuenz, Markus Reindl, Maria Morandell, Thomas Berger, Erich Schmutzhard, Christian Eggers

**Affiliations:** 1 Department of Neurology, Innsbruck Medical University, Innsbruck, Austria; 2 Department of Neurology, Krankenhaus Barmherzige Brüder, Linz, Austria

## Abstract

**Background:**

Neuroinflammation and demyelination have been suggested as mechanisms causing HIV-1 associated neurocognitive disorder (HAND). This cross-sectional cohort study explores the potential role of antibodies to myelin oligodendrocyte glycoprotein (MOG), a putative autoantigen in multiple sclerosis, in the pathogenesis of HAND.

**Methods:**

IgG antibodies against MOG were measured by ELISA in sera and cerebrospinal fluid (CSF) of 65 HIV-positive patients with HAND (n = 14), cerebral opportunistic infections (HIVOI, n = 25), primary HIV infection (HIVM, n = 5) and asymptomatic patients (HIVasy, n = 21). As control group HIV-negative patients with bacterial or viral CNS infections (OIND, n = 18) and other neurological diseases (OND, n = 22) were included. In a subset of HAND patients MOG antibodies were determined before and during antiviral therapy.

**Results:**

In serum, significantly higher MOG antibody titers were observed in HAND compared to OND patients. In CSF, significantly higher antibody titers were observed in HAND and HIVOI patients compared to HIVasy and OND patients and in OIND compared to OND patients. CSF anti-MOG antibodies showed a high sensitivity and specificity (85.7% and 76.2%) for discriminating patients with active HAND from asymptomatic HIV patients. MOG immunopositive HAND patients performed significantly worse on the HIV dementia scale and showed higher viral load in CSF. In longitudinally studied HAND patients, sustained antibody response was noted despite successful clearance of viral RNA.

**Conclusions:**

Persistence of MOG antibodies despite viral clearance in a high percentage of HAND patients suggests ongoing neuroinflammation, possibly preventing recovery from HAND.

## Background

HIV encephalopathy (HIVE) leads to dementia and motor disorder and is the major direct central nervous system (CNS) manifestation of advanced HIV-1 infection. Since the availability of combination antiretroviral therapy (cART) its incidence has decreased, but to a lesser extent than the incidence of extra-cerebral AIDS-manifestations [1]. With the increasing life expectancy of HIV-infected individuals the prevalence of HIV associated neurocognitive disorder (HAND) has risen to 20-50% [2]. While it is generally accepted that HAND is treatable, the extent and sustainability of the effects of cART on cerebral functioning are still unclear. There is accumulating evidence of chronically progressive and, at times, fluctuating cognitive impairment in patients with effective cART in terms of suppression of plasma viral load [3,4], compatible with the notion of quiescent and active disease [5]. While HIV is the *agens movens *of HIVE, it does not damage neuronal cells directly. Rather, a plethora of cellular and molecular immunological mechanisms leads to neurological dysfunction [6]. Demyelination has early been recognized as a feature in the pathological and radiological appearance of HIVE [7,8], and cases with early-stage HIV infection clinically mimicking multiple sclerosis (MS) have been described [9]. Myelin breakdown products and antibodies against them have been implicated in this context. In particular myelin basic protein has been suggested to be of prognostic significance [10,11].

Another myelin protein that has been extensively studied in MS is myelin oligodendrocyte glycoprotein (MOG) [12]. MOG is a quantitatively minor type I transmembrane protein exclusively expressed in the CNS, and its extracellular domain has been identified as a main target for immune responses in experimental allergic encephalitis (EAE), an animal model for MS [13]. However, in humans antibodies against MOG are mainly found in patients with acute demyelinating encephalomyelitis (ADEM) or childhood MS [14-16] whereas their value in adult MS is still under debate [17]. Anti-MOG antibodies are also detected in infectious diseases of the CNS [18], and their presence correlates with the titers of antibodies to Epstein Barr Virus (EBV) [19]. To our knowledge, this cross-sectional cohort study is the first to evaluate the potential role of MOG antibodies in cerebrospinal fluid (CSF) and serum of patients with HIV as markers for disease course and response to antiviral therapy.

## Methods

### Patient characteristics

Within a six-years period 65 consecutive HIV patients were recruited at the University Hospital Hamburg, Germany. The primary care-giving physicians of the Medical Department presented the patients to the Neurological Department for the clinical and diagnostic workup for potential neurological disease, and a proportion of subjects took part in an observational study for CNS manifestations of HIV infection. The visits were done by a single neurologist (CE) experienced in the treatment of HIV infection. Patients underwent lumbar puncture (LP) for the evaluation of neurological manifestations of HIV infection or as part of the observational study. In subjects with longitudinal sampling LP was performed prior to initiation or change of therapy and at variable intervals thereafter with a minimum of one follow-up lumbar puncture during cART. Peripheral blood samples were obtained in parallel with lumbar puncture. Cognitive impairment was quantified by the HIV dementia scale (HDS) [20]. HIV patients were classified in four groups for further analyses: The "HIV associated neurocognitive disorder group" (HAND) consisting of 14 patients with the typical clinical phenotype of HIV associated neurocognitive disorder and exclusion of differential diagnoses, the "HIV with opportunistic infection group" (HIVOI) comprising 25 patients with a cerebral opportunistic infection (CNS toxoplasmosis n = 10; progressive multifocal leukoencephalopathy n = 6, cryptococcal meningitis n = 5, neurosyphilis n = 2, cytomegalovirus encephalitis n = 2), the "HIV meningitis group" (HIVM) including 5 patients with aseptic meningitis in the context of serologically proven primary HIV infection, and the "HIV asymptomatic group" (HIVasy) with 21 HIV-infected patients in whom neurological involvement was excluded by clinical, laboratory (including spinal tap) and imaging findings.

HIV-negative controls with normal cognitive function were recruited at the Department of Neurology, Innsbruck Medical University. Subjects with other infectious neurological diseases (OIND) and non-inflammatory other neurological diseases (OND) served as another control group. The 18 OIND patients suffered from bacterial meningitis (pneumococci n = 5, meningococci n = 5, staphylococcus aureus n = 1, beta-hemolytic streptococci n = 1, and undetermined microorganism n = 2) and herpes simplex-1 meningoencephalitis (n = 4). The 22 OND patients were diagnosed with pseudotumor cerebri, migraine, psychogenic neurological symptoms, sinus venous thrombosis, vascular leukoencephalopathy, herniated vertebral disc, transient ischemic attack, spastic paraparesis, multiple system atrophy, neuropathic pain syndrome, cerebellar infarction, brain tumor, focal dystonia, and ischemic transverse spinal cord syndrome. The study was approved by the respective ethics committees of Hamburg and Innsbruck Medical University, and all patients declared their informed consent. CSF cytology, CSF white and red blood cell count, total protein, CSF to serum albumin quotient (Qalb i.e. [Alb]CSF/[Alb]Ser × 1000), and intrathecal production of immunoglobulin G (IgG-index) was analyzed in every subject. Samples with a red blood cell count of more than 20/μl in the CSF were excluded.

### Antibody analysis

The presence of IgG antibodies to recombinant human MOG extracellular Ig domain (amino acids 1-125) produced in E. coli bacteria was determined by ELISA, as described previously [19,21]. In serum, a value of 0.6 optical density (OD) units for anti-MOG IgG was defined as cutoff for a positive result, as derived from a pool of external reference samples from healthy individuals [21].

### Statistical analyses

Patient characteristics were compared between groups by ANOVA (age), Kruskal-Wallis test (CSF white blood cell count, Qalb, IgG-index), Chi-square test (sex, presence of MOG antibodies) or Wilcoxon-rank-sum test (HDS) depending on the data type and distribution. Optical density values for MOG antibodies were compared between groups by Kruskal-Wallis test, post-hoc analysis was done by Dunn's Multiple Comparison Test. Repeated measures data from longitudinally studied subjects (CSF/plasma viral load, CD4+ cell counts and OD values for MOG antibodies) was logarithmically transformed and modeled by generalized estimation equations (GEE). For evaluating the discriminatory power of MOG antibodies receiver operator characteristic (ROC) curves were drawn and cut-off points were chosen equally weighting sensitivity and specificity. Calculations were performed with SPSS 16 (SPSS, Chicago, IL, USA) and GraphPad Prism 5.00 (GraphPad Software, San Diego, CA, USA).

## Results

A total of 65 subjects with HIV infection were included in this study. For the assignment to groups see Methods. Demographic, clinical and CSF data of the respective patient groups are given in table 1.

**Table 1:** Patient characteristics

	HAND	HIVOI	HIVM	HIVasy	OIND	OND	p-value
	
n	14	25	5	21	18	22	
age (mean and STD)	45.2 (11.9)	41.5 (10.4)	33.8 (12)	35.9 (11.2)	51.6 (19.2)	42.6 (12.4)	0.007
female gender (%)	3 (21.4%)	5 (20.0%)	2 (40.0%)	0 (0.0%)	9 (50.0%)	15 (65.2%)	< 0.001
							
CSF cells/μl (mean and STD)	5.9 (7.5)	12.9 (15.1)	65.9 (94.9)	2.3 (2.3)	2313 (3051)	2 (2)	< 0.001
IgG-index (mean and STD)	0.97 (0.39)	0.88 (0.27)	0.60 (0.14)	0.63 (0.15)	0.82 (0.48)	0.48 (0.07)	< 0.001
Qalb (mean and STD)	9.3 (3.9)	10.8 (6.6)	12.9 (8.8)	6.8 (5.7)	47.3 (48.8)	5.6 (2.3)	< 0.001
							
MOG IgG serum positivity (%)	7 (50.0%)	10 (40.0%)	1 (20.0%)	1 (4.8%)	6 (33.3%)	1 (4.5%)	0.003

### MOG antibody response is elevated in HIV patients with CNS involvement

The highest percentage of patients positive for anti-MOG antibodies in serum was observed in the HAND group (50.0%, table 1). In the HIVOI and OIND group 40.0% and 33.3% of patients were anti-MOG antibody positive, respectively. Only one patient (20.0%) with HIVM, one patient (4.5%) in the OND group and one patient (4.8%) of the HIVasy group were positive for anti-MOG antibodies. This difference was statistically highly significant (p = 0.003).

**Figure 1:**
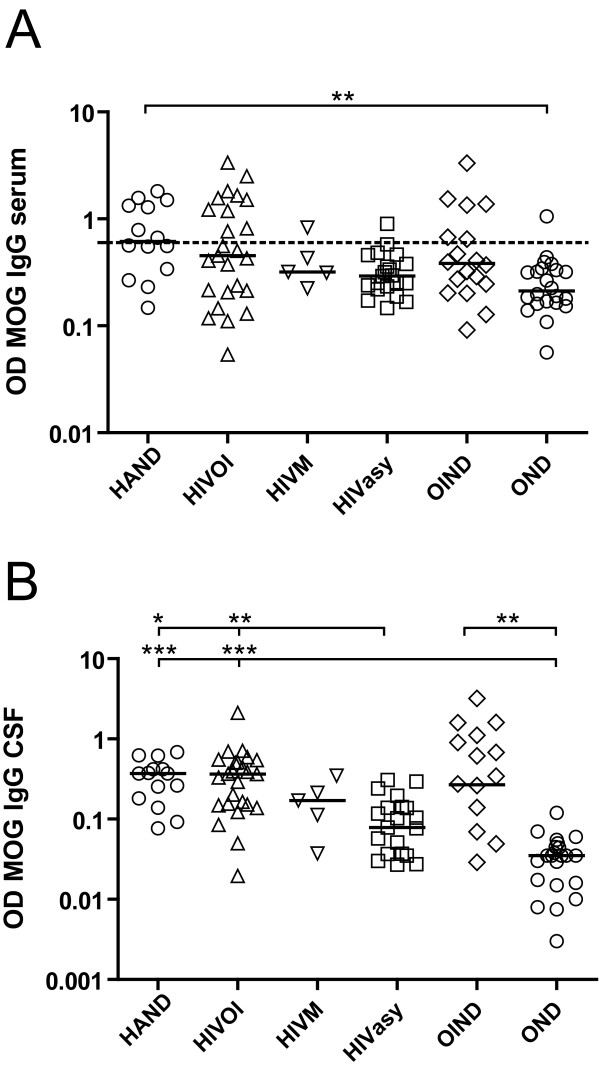
**IgG antibody response to MOG in serum and CSF**. Serum (A) and CSF (B) IgG antibody response to MOG in patients with HIV associated neurocognitive disorder (HAND), HIV patients with opportunistic infections of the CNS (HIVOI), HIV meningitis (HIVM), asymptomatic HIV infection (HIVasy), other infectious neurological diseases (OIND) and other neurological diseases (OND). Kruskal Wallis test (A: p < 0.01, B: p < 0.001). Dunn's post hoc test yielded a significant difference between HAND and OND patients (p < 0.01, A). The dotted line indicates a cut-off value of 0.6 OD, which is considered as serum immunopositivity for MOG IgG. In CSF samples Dunn's post hoc test yielded a significant difference of HAND and HIVOI patients compared to HIVasy (p < 0.05 and p < 0.01) and OND patients (both p < 0.001) and OIND compared to OND patients (p < 0.01). Data is shown on a logarithmic scale; the horizontal bar represents the median.*, p < 0.05; **, p < 0.01; ***, p < 0.001.

The concentrations of anti-MOG IgG, as determined by the OD values, were also significantly different between groups (p = 0.006). Post hoc analyses yielded significantly higher MOG-IgG OD values in the HAND group compared to the OND group (p < 0.01, figure 1A).

Unlike for serum, there is no established cut-off value for antibody reactivity in CSF. Therefore OD values were compared, yielding a highly significant difference between groups (p < 0.001, figure 1B). Post hoc analyses showed significantly higher MOG-IgG OD values in HAND and HIVOI patients compared to HIVasy (p < 0.05 and p < 0.01) and OND (both p < 0.001) patients. In OIND patients also higher MOG-IgG OD values were observed compared to OND patients (p < 0.01).

### MOG antibodies discriminate HAND from asymptomatic HIV patients

In order to further elaborate the clinical significance of anti-MOG antibodies, receiver operator characteristic (ROC) curves where drawn for discriminating HAND from asymptomatic HIV patients. The area under the curve (AUC) was 0.881 (confidence interval 0.766-0.996) for CSF anti-MOG IgG antibodies and 0.789 (confidence interval 0.617-0.961) for serum anti-MOG IgG antibodies. The AUC for the IgG-index was 0.810 and 0.806 for the albumin quotient, respectively. In CSF, a cut-off value of 0.14 yielded a sensitivity of 85.7% and a specificity of 76.2% for discriminating HIV positive patients with active HAND from asymptomatic HIV patients. In serum, a cut-off value of 0.27 yielded a sensitivity of 85.7% and a specificity of 47.6%.

### MOG antibodies correlate with grade of dementia in HAND

In eight patients with HAND the HDS was assessed during a total of 17 visits.

The baseline HDS result was significantly more impaired in subjects with detectable anti-MOG serum IgG antibodies (median HDS = 2, range = 2-3) as compared to the MOG seronegative subjects (median HDS = 16, range = 10-16, p = 0.021), i.e. these patients were more severely demented.

### MOG antibodies are associated with CSF viral load

**Figure 2:**
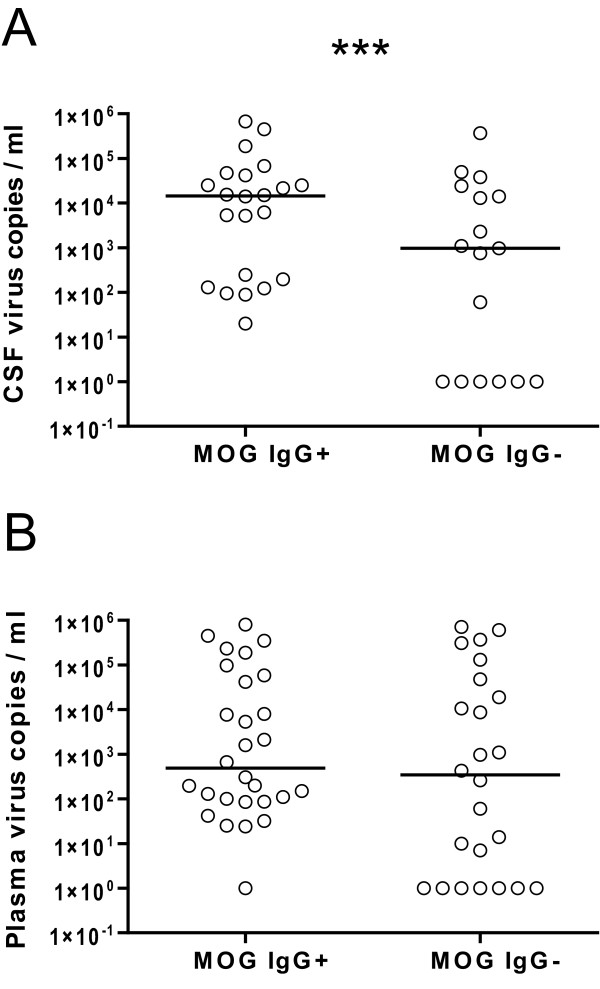
**Viral load in CSF and plasma samples**. Viral load in CSF (A) and plasma samples (B) (virus copies per milliliter) stratified for positive serum anti-MOG IgG antibodies. Data is shown on a logarithmic scale, samples below the detection limit are shown as 1 virus copy/ml. The horizontal bar represents the median. Repeated measures data was modeled by generalized estimation equations. ***, p < 0.001.

**Figure 3:**
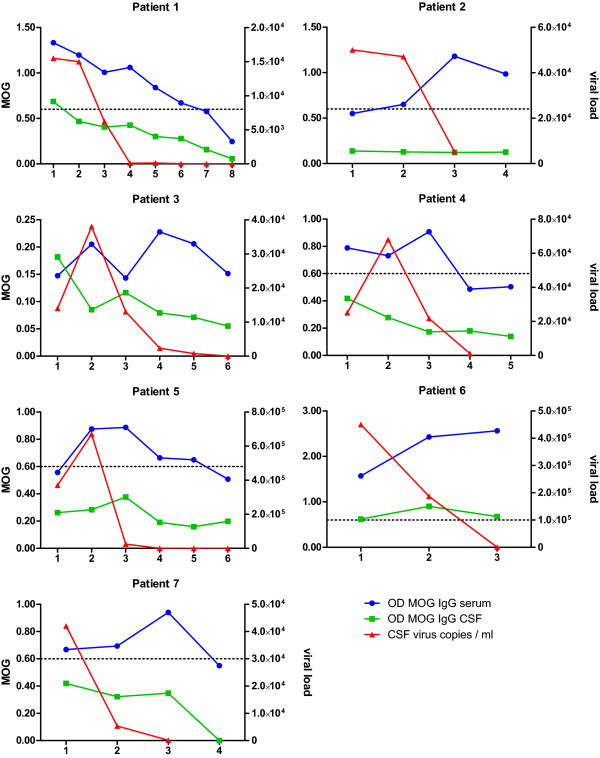
**IgG antibody response to MOG in longitudinally studied patients**. Serum anti-MOG IgG antibody responses in 7 patients with HIV associated neurocognitive disorder (HAND) documented follow-up visits and available CSF viral load data. The dotted line indicates a cut-off value of 0.6 OD, which is considered as serum immunopositivity for MOG IgG. Groupwise repeated measures data was modeled by generalized estimation equations and showed a highly significant effect (p < 0.001) of time of sampling on the concentration of anti-MOG antibodies.

Plasma and CSF viral load was available for 13 patients during a total of 40 visits. Viral load in CSF was significantly higher in anti-MOG antibody positive than in anti-MOG negative serum samples (p < 0.001, GEE, figure 2A, figure 3). Viral load in plasma, however, was not significantly different between anti-MOG antibody positive and negative serum samples (figure 2B). In addition, there was no association between CD4^+ ^cell counts and MOG serum antibody positivity (data not shown).

### MOG antibodies in longitudinally studied subjects

All patients with HAND were initially cART naïve. Ten patients underwent documented follow up visits with repeated CSF/blood sampling. In seven patients longitudinal viral load data were available. Anti-MOG antibodies and CSF viral load in these patients are shown in figure 3. Most patients showed sustained anti-MOG IgG response in serum despite successful clearance of viral RNA from CSF (figure 3). Initiation of cART was followed by an initial increase in the OD values in some patients. With continuing treatment anti-MOG antibodies decreased and became negative after clearance of viral RNA in four patients. The mean time interval to seroconversion in these patients was 447 days (range 179-782 days). The time dependent decrease of anti-MOG antibodies after initiation of cART was supported by a statistically highly significant effect of time of sampling on antibody concentration in the GEE model (p < 0.001). The mean time interval between the visits was 128 days (range 13-673 days). Only three out of 10 patients did never show antibodies against MOG.

### Case report: increasing leukoencephalopathy under successful cART

**Figure 4:**
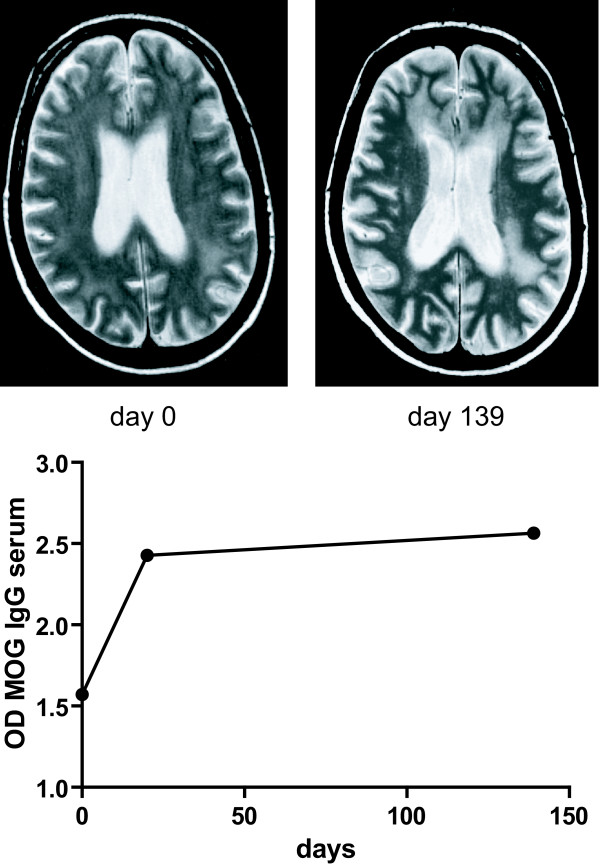
**Case report**. Case report: increasing leukoencephalopathy under successful cART. MR scans and OD values for MOG IgG of one patient with HAND are shown.

MRI scans and OD values for MOG IgG antibodies of one patient with HIV associated dementia (HAD) are shown in figure 4. This 35 year old male patient with HAD initially presented with severe dementia (HDS of 2), a CD4+ cell count of 145/μl and a plasma and CSF viral load of 58000 and 450000 viral copies/ml, respectively. After initiation of cART, rapid elimination of the virus in plasma but considerably delayed clearance from CSF was noted. Although the patient had clinically improved 20 weeks after the first visit, the MRI scan showed a deterioration of leukoencephalopathy paralleled by a sharp increase of OD values for MOG IgG antibodies.

## Discussion

The pathophysiology of HIVE remains incompletely understood. While in the cART era the incidence of AIDS-defining conditions showed a dramatic decrease, AIDS dementia complex is still observed in a comparably high percentage of patients and despite successful viral clearance [22]. Ongoing autoimmune mechanisms have been suggested as an explanation for this discrepancy [6]. Besides activation of brain microglia, complement-mediated mechanisms have been implicated in the development of neurological dysfunction [23,24]. Complement is readily activated by antibodies binding to cellular epitopes. With respect to autoimmune phenomena, CSF antibody responses to CNS autoantigens such as myelin basic protein (MBP) have been demonstrated [11,25]. These responses seemed to be more prominent in the CSF of neurologically symptomatic HIV subjects. However an association with serum antibodies was not found [25,26].

In our study, we found higher anti-MOG reactivity in CSF and serum of patients with HAND as compared to OND patients. For autoantibodies to CNS antigens it is generally accepted that some degree of brain tissue damage as well as an increased blood-brain-barrier permeability is needed for the antigen to be presented to the immune system. Such a lesion to cerebral tissue may be assumed in acute HIV-associated opportunistic CNS-infections as well as in non-HIV-associated meningitis/encephalitis (the groups HIVOI and OIND). Therefore, our finding that anti-MOG antibodies were highest in the serum and CSF of patients with HAND is somewhat surprising, as accepted signs of inflammation such as CSF pleocytosis and contrast enhancement on MRI are conspicuously absent in HIVE.

A significant finding is the higher values of CSF and (less so) serum MOG antibodies in the HAND group as compared to the group of asymptomatic HIV subjects. As HIV invades the CNS in every infected subject from early on, the presence of the virus on its own cannot account for the higher MOG antibody titers in HAND subjects. In addition, our finding of higher MOG antibody titers in more demented patients adds to the impression that anti-MOG antibody formation, in the absence of grossly destructive inflammation, may be a relevant biomarker for HAND.

Another finding of clinical importance is the high discriminatory power of CSF anti-MOG antibodies (AUC = 0.881) compared to other routine CSF parameters in ROC analyses. Importantly, a cut-off value of 0.14 for CSF anti-MOG antibodies was highly sensitive and specific in distinguishing patients with active HAND from asymptomatic HIV patients. Since opportunistic infections and HIV-associated meningitis are easily detected by an elevated CSF cell count, elevated CSF anti-MOG antibodies could aid the clinician in the sometimes difficult diagnosis of HAND.

Our result of an association of anti-MOG antibody titers with the viral load in CSF, but not plasma, suggests that the immunological activity in the CNS may be dissociated from immune processes in the systemic compartment. This corresponds with the well recognized correlation of HIV dementia with CSF, but not plasma viral load [27], and the delayed suppression of CSF viral replication upon initiation of cART in HAND patients [28].

With successful treatment of a microbial assault to the brain, as represented in our study by suppression of HIV production in the CSF and plasma, one would expect the stimulus for the production of autoantibodies to eventually disappear. Accordingly, in microbial meningitis and systemic inflammatory diseases anti-MOG antibodies have been shown to be transient [18]. In this respect, the persistence of MOG antibodies over months to years in six of our patients and, even more so, the emergence of MOG antibodies in one patient only after initiation of cART is of particular interest.

A related observation was the increase of anti-MOG antibodies in the early samples after initiation of cART despite decrease of the plasma and (to a lesser extent) CSF viral load. This was particularly pronounced in one patient in whom follow-up brain MRI showed an intermittent marked increase in white matter lesions in contrast to clinical improvement of his HAND.

The discordance between anti-MOG antibodies and viral suppression in many patients suggests that immunological mechanisms are relatively independent from virus concentrations. Eden et al. showed sustained immunologic activity as judged by persistently elevated CSF neopterin levels in HIV-positive individuals with long-term suppression of plasma and CSF viral load [29].

In the patient presented as case vignette the progressive white matter lesions in parallel to increasing anti-MOG antibodies might be regarded as an immune reconstitution phenomenon in response to CNS autoantigens similar to the clinically overt immune reconstitution syndromes that have been described in HIV patients with opportunistic infections upon start of treatment [30].

This study has some limitations. First, the retrospective collection of some of the data may have introduced bias. While HAND, HIVOI and the OND group showed comparable age distribution, patients with primary HIV infection and asymptomatic HIV patients were younger and OIND patients were older. Although an age-dependent effect of immune response to myelin components cannot be excluded, it has so far not been observed by others [18,21]. In addition, the main difference in serum anti-MOG activity was observed between HAND and OND patients who were well matched. Hence, we are confident that this difference should not adversely influence the interpretation of the data. Second due to the limited number of included HIV positive patients, the study was underpowered to detect differences among subgroups of HAND patients. Further studies will have to address the question if anti-MOG antibodies are able to differentiate HAND patient with asymptomatic neurocognitive impairment or mild neurocognitive disorder from neurologically asymptomatic patients as it was the case in patients with HAD in the current study. Third, in MS the role of MOG antibodies is still under debate, and a number of recent studies suggest that antibodies to native MOG might only be pathogenic in patients with acute disseminated encephalomyelitis (ADEM) or childhood MS [14-16], whereas they are an epiphenomenon in "classical MS" that might be related to ongoing tissue damage [18,31] or EBV infection [19]. In this study we have used recombinant human MOG extracellular Ig domain (amino acids 1-125) produced in E. coli bacteria to determine anti-MOG antibodies by ELISA. This recombinant protein was refolded after purification [32] and has been widely used for immunological studies in patients and animal models (reviewed by [13] and [12]) and for the determination of the three-dimensional structure of MOG [33]. Recent data indicate that pathogenic antibodies recognize the physiological membrane topology and glycosylation pattern of MOG [34], whereas antibodies to the MOG preparation used in the current study reflect CNS inflammation and a secondary response to myelin debris rather than a pathogenic antibody response. However, the exact role of antibodies to surface exposed conformational epitopes and glycosylated MOG in HAND require further studies.

## Conclusions

In conclusion, our study demonstrates high rates of seropositivity and high titers of CSF antibodies to MOG in patients suffering from HAND as compared to asymptomatic HIV-infected and unifected subjects. In a proportion of HAND patients these autoantibodies persist or even develop upon initiation of cART with successful clearance of viral RNA from CSF, suggesting that antibody-mediated mechanisms of brain damage might play a role in the neuropathogenesis of AIDS. In addition, determination of serum anti-MOG antibodies might be a new non-invasive marker for the discrimination of active versus quiescent neuroinflammation in the context of HIVE.

## Competing interests

There is neither a relationship nor a support which might be perceived as constituting a conflict of interest of any of the authors.

## Authors' contributions

PL, ES and CE had the idea and designed the study. BK and MM performed the sample analyses. MR supervised all laboratory analyses. PL and MR performed the data analyses. TB helped with the design of the study and the interpretation of the results. PL, ES and TB recruited control patients, CE recruited all HIV patients. PL and CE drafted the manuscript. All authors have read and approved the final version of the manuscript.
